# Modulatory Effects of Exogenously Applied Polyamines on Postharvest Physiology, Antioxidant System and Shelf Life of Fruits: A Review

**DOI:** 10.3390/ijms18081789

**Published:** 2017-08-17

**Authors:** Sunil Sharma, Sunil Pareek, Narashans Alok Sagar, Daniel Valero, Maria Serrano

**Affiliations:** 1 Department of Agriculture and Environmental Sciences, National Institute of Food Technology Entrepreneurship and Management, Kundli, Sonepat, Haryana 131028, India; sharma.agribiotechnology@gmail.com (S.S.); sunil_ciah@yahoo.co.in (S.P.); narashans.alok@gmail.com (N.A.S.); 2 Department Food Technology, University Miguel Hernández, Orihuela, Alicante 03000 Spain; daniel.valero@umh.es; 3 Department Applied Biology, University Miguel Hernández, Orihuela, Alicante 03000 Spain

**Keywords:** polyamines, putrescine, spermine, spermidine, physiology, respiration, ethylene, chilling injury, shelf life, antioxidants, encoding genes, *S*-adenosyl methionine

## Abstract

Polyamines (PAs) are natural compounds involved in many growth and developmental processes in plants, and, specifically in fruits, play a vital role regulating its development, ripening and senescence processes. Putrescine (PUT), spermine (SPE), and spermidine (SPD) are prominent PAs applied exogenously to extend shelf life of fruits. They also originate endogenously during developmental phases of horticultural crops and simultaneously affect the quality attributes and shelf life. Their anti-ethylene nature is being exploited to enhance the shelf life when exogenously applied on fruits. In growth and development of fruits, PA levels generally fall, which marks the beginning of senescence at postharvest phase. PUT, SPE and SPD treatments are being applied during postharvest phase to prolong the shelf life. They enhance the shelf life of fruits by reducing respiration rate, ethylene release and enhance firmness and quality attributes in fruits. PAs have a mitigating impact on biotic and abiotic stresses including chilling injury (CI) in tropical and sub-tropical fruits. PAs are environment friendly in nature and are biodegradable without showing any negative effect on environment. Biotechnological interventions by using chimeric gene constructs of PA encoding genes has boosted the research to develop transgenic fruits and vegetables which would possess inherent or in situ mechanism of enhanced biosynthesis of PAs at different stages of development and thereby will enhance the shelf life and quality in fruits. Internal and external quality attributes of fruits are improved by modulation of antioxidant system and by strengthening biophysical morphology of fruits by electrostatic interaction between PAs and phospholipids in the cell wall.

## 1. Introduction

Polyamines (PAs) are the aliphatic, cationic organic compounds participating in the growth and development of living organisms. Naturally found PAs include putrescine (PUT), spermine (SPE) and spermidine (SPD). PAs exist both in free and conjugated forms in plants [1]. The amine functional group in these compounds leads to their varying functional properties in the physiology of living organisms and plant systems.

The appearance of senescence in the fruits after the harvest devalues market potential. Ethylene production remains at peak after the harvest which causes shrivelling in the outer peel of fruit and diminishes its flavour and other quality attributes and ultimately the shelf life. PAs restricts the transcriptional synthesis of ethylene and 1-aminocyclopropane-1-carboxylic acid (ACC)-synthase and thereby blocks the activity of ACC oxidase by removal of free superoxide radicals which are essentially required for the conversion of ACC to ethylene [2]. It is already reported that PAs are bounded to cell wall which imparts strength to cell wall [3]. PAs imparts the firmness to the fruit under storage by developing the electrostatic bonding with the negatively charged pectic substances in the cell walls of fruits [4]. Therefore, when PAs are applied exogenously, they play a vital role in enhancing the shelf life of fruits. This has been discussed in few reviews but with a lack of comprehensive approach regarding the postharvest physiological roles of PAs in enhancing the shelf life. Early reviews on PAs have focussed on the role in mitigating chilling injury (CI) in fruits and vegetables [5], stress tolerance under diverse stresses [6,7], PA metabolism and its role in countering abiotic stresses [8] and only one review [9] is found which has discussed the role of different PAs with the perspective of improving the shelf life of fruits. Review has discussed the changes in the levels of PAs in climacteric and non-climacteric fruits during the postharvest phase. It has also briefly discussed about the effects of PAs on fruit quality when exogenously applied and when PAs are produced endogenously [9]. After publishing this review, various advancements have been made in PA research, and much information made available during the last one and half decade. Therefore, the present paper reviewed the effect of exogenously applied PAs on physiology and shelf life of fruits (Table 1). Besides this, we reviewed the application of biotechnological tools by way of developing in situ mechanism for over-expression of genes encoding PAs and their impact on the quality of tomato fruit. Over-expression of genes encoding PAs in tomato has improved the quality attributes such as juiciness, and accumulation of phytonutrients and the other quality attributes [10,11]. This article analysed the diverse role of exogenous application of PAs and over expression of genes encoding PAs and is enlightening the scientific research to be followed in this direction in order to enhance the shelf life. The in situ over-expression of such genes encoding PAs will provide us the inherent mechanism to develop fruits having prolonged shelf life and low CI under normal and cold storage conditions, respectively.

## 2. Biosynthetic Pathways of Important Polyamines

**Figure 1:**
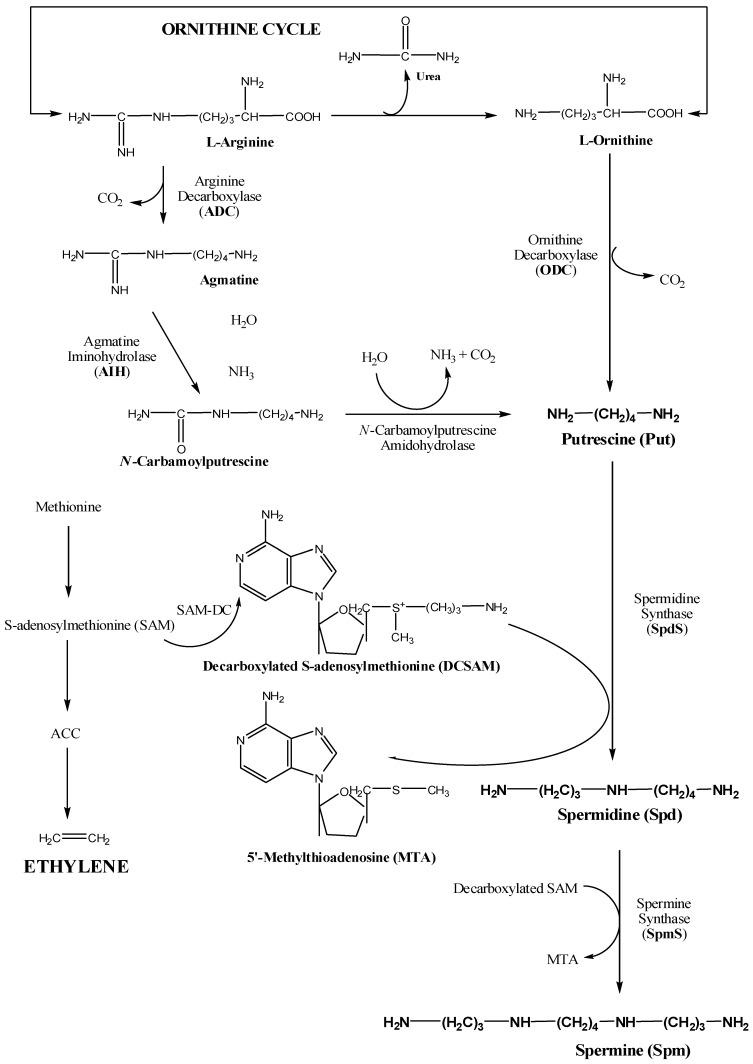
Biosynthetic pathway of three naturally synthesised prominent polyamines: Spd, Spermidine; Spe, Spermine; Pu, Putrescine. Precursors involved in biosynthesis: l-Arginine, l-Ornithine; *N*-carbamoylputrescine; Agmatine; Methionine; SAM, *S*-adenosylmethionine; ACC, 1-aminocyclopropane-1-carboxylic acid. Enzymes involved in the pathway: ADC, Arginine decarboxylase; ODC, Ornithine decarboxylase; AIH, Agmatineimino hydrolase; *N*-CarbamoylputrescineAminohydrolase; SpdS, Spermidine synthase;SpmS, Spermine synthase.

Figure 1 described the biosynthetic pathway of naturally existing PAs i.e., PUT, SPE and SPD. Biosynthesis of PAs was explained in diverse sets of organisms viz. plants, bacteria, mammals and fungi [12,13,14,15]. Amino acids l-arginine and l-ornithine act as primary precursors for biosynthesis of PUT and indirectly for SPE and SPD as well. Decarboxylation of l-ornithine by ornithine decarboxylase (ODC) synthesises PUT, whereas the decarboxylation of l-arginine by arginine decarboxylase synthesises an intermediate PA called agmatine which in turn is acted upon by agmatimeiminohydrolase (AIH) to form *N*-carbamoyl putrescine. *N*-carbamoyl putrescine is acted upon by *N*-carbamoyl putrescine aminohydrolase to form PUT. The conversion of PUT by spermidine synthase (SpdS) directly synthesises SPD and consequentially the SPD is acted upon by spermine synthase (SpeS) to synthesise SPE. Besides this, *S*-adenosyl methionine (SAM) whose precursor is methionine acts as a balancing intermediate to facilitate the synthesis of ethylene and at the same time facilitates the biosynthesis of natural PAs, SPE and SPD. In general, the activity of *S*-adenosyl methionine decarboxylase is one of the steps which facilitates the synthesis of SPE and SPD. The particular characteristics of *S*-adenosyl methionine decarboxylase encoding gene has made it to be widely applied in the transgenic crops for facilitating the in situ biosynthesis of PAs which ultimately controls the ethylene biosynthesis in coherence with synthesis of PAs particularly SPE and SPD to improve the quality attributes of fruits.

## 3. Effect of Polyamines on Postharvest Physiology, Antioxidant System and Biochemical Attributes of Some Fruits

PAs make links with pectin and cell wall components [16] with anions such as phospholipids of the cell membranes [17]. Free forms of PAs act as anti-senescent agents, reduce rate of respiration, delay ethylene production, retard colour changes, increase the fruit firmness, induce mechanical resistance and reduce chilling symptoms [9]. Many studies on the effects of postharvest exogenous application of PAs were conducted for enhancing the storage life of fruits, e.g., in apricot [18,19,20], grapes [21], kiwifruit [22,23], mango [24,25,26], plums [27,28,29], pomegranate [30,31,32], strawberry [33], and zucchini [34]. This review comprehensively covers the effect of PAs on maintaining the shelf life of fruits when applied exogenously under postharvest storage conditions.

### 3.1. Effect on Shelf Life and Fruit Quality

The anti-ethylene nature of the PAs led them to act as shelf life enhancing amines by affecting the diverse physiological processes under the storage conditions and enhancing the shelf life of fruits (Table 1). PAs application in climacteric and non-climacteric fruits were reported to reduce the mechanical damage, CI and enhanced shelf life of fruits along with delayed colour changes [5,27]. In apricots, treatment with PUT (1 mM) led to the improvement in shelf life of undamaged and the damaged fruits, by reducing ethylene evolution and respiration rates and mitigated the mechanically damaged bruised zones [18]. Similar results were reported in mango cv. Kensington Pride where PUT (2 mM) when exogenously applied led to enhancement in shelf life of fruits [24]. Exogenous application of PUT (1 mM) on “Hayward” kiwifruit showed reduced rate of respiration, ethylene release and subsequent increase in shelf life and firmness of flesh and maintained the overall quality attributes of fruit. PUT (1 mM) when applied to “Mauricio” apricots and subjected to mechanical damage by the application force of around 25 N and then kept under storage for 6 days at 10 °C improved the quality and shelf life of fruits by remarkably decreasing the weight loss, color change, ethylene emission and respiration rates of the treated fruit [18]. Similarly, fruits oftwo cultivars of Iranian apricots, namely “Lasgerdi” and “Shahrodi”, when treated with varied concentrations of PUT, 1.0, 2.0, 3.0 and 4.0 mM, followed by packaging in covered containers made of polyethylene and stored (4 °C, 95% RH) for 20 days .PUT 4 mM was found to be effective leading to reduction in weight loss, followed by maintenance of firmness and simultaneous modulation of the antioxidant system, total phenol content (TPC), ascorbic acid (AA) and finally improved shelf life and quality of fruits [20]. In “Flame Seedless” grapes, both PUT and SPD at 0.5 mM each maintained the healthy green rachis up to 60 days of storage whereas higher dose of PAs proved to be detrimental leading to weight loss and browning of rachis and therefore affecting the quality and shelf life of grapes [21]. Exogenous SPE dip treatment in “Flame Seedless” grape was also found effective in maintaining fruit quality and in extending the shelf life [35]. Exogenous application of PAs viz. SPE and SPD was applied to kiwifruit cv. Allison. The immersion method was followed to apply PAs in kiwifruits under ambient conditions at 22 ± 4 °C and RH 65 ± 5% [23]. Four plum cultivars (“Santa Rosa”, “Black Star”, “Black Diamond” and “Golden Japan”) when exogenously treated with PUT (1 mM) followed by their storage at 20 °C resulted in enhancement of shelf life and improved quality attributes of fruits [28]. The treatment of PUT and carnauba wax in the ratio of 1:10 applied to “Mridula” pomegranates before the cold storage at 2 °C reported to enhance the shelf life of fruits by reducing the CI and maintained the fruit quality by mitigating discoloration, strengthening the firmness of fruit peel without any weight loss. Therefore, PUT in addition to carnauba wax resulted in enhancing the shelf life of fruits followed by an effective reduction in respiration rates and ethylene release rate. Higher juice recovery was obtained in treated fruits, in which the applied combination of PUT and carnauba wax contributed to maintenance of fruit quality attributes and high recovery of juice from arils in comparison to control treatment [32]. The immersion of fruit of strawberry cv. “Selva” in PUT aqueous solution at different concentrations of 0.3, 0.5, 1 or 2 mM under 5 °C temperature showed reduced incidence of fungal infection which led to the improvement in the appearance of fruit by inducing an increase in firmness of fruit, but no significant effect on pH, TSS and TA was reported. Taste panel reported the improvement in quality attributes of strawberry [33]. Therefore, PA application led to enhancement in the shelf life of fruits along with the maintenance of quality attributes due to the aggregate effect of reduction in ethylene release, enhancement in firmness of fruit, suppressive effect on cell wall degrading enzymes and positive modulation of antioxidative enzyme machinery.

### 3.2. Effect on Postharvest Physiology

#### 3.2.1. Effect on Weight Loss

Weight loss after harvest is a common phenomenon which ultimately deteriorates fruit quality. PAs have potential to reduce weight loss during postharvest (Table 1). Application of PUT (0.0, 1.0, 2.0 or 3.0 mM) on mango cv. Langra significantly affected shelf life and quality attributes. PUT (2.0 mM) was found to be best concentration which led to reduction in loss of physiological weight and percentage of spoilage in comparison to the untreated fruits [25]. Application of low concentration of SPM (0.5 mM) and higher concentration of PUT (1 mM) or SPD (1 mM) in mango cv. Kensington Pride were found effective in reducing weight loss in comparison to untreated fruits under storage [36]. PUT (1 mM) treatment on the damaged apricots led to a significant reduction in weight loss, rate of respiration, in both damaged and control fruits [18].

Maximum 17% weight loss was observed in control pomegranate fruits, whereas pre-storage treatment of PAs has led to 13–15% reduction in weight [30]. Combination of PUT (2 mM) with carnauba wax in the ratio of 1:10 in pomegranates led to ~10% reduction in weight loss as compare to untreated fruits, which showed a ~17% of weight loss after 60 days of storage [32]. Therefore, the study showed the efficiency of the combinatorial effect of PAs with the carnauba wax which acted as a physical barrier to slow down the ripening metabolism in fruits. However, postharvest application of PUT (0.3, 0.5, 1.0, or 2 mM) on strawberry cv. Selva stored at 5 °C showed no significant differences on weight loss between control and treated fruit [33].

#### 3.2.2. Effect on Fruit Firmness

Pectic substances play an important role in providing firmness to fruits, which is one of the most important fruit quality parameters. The diverse set of enzymes plays a vital role in reducing or degrading the firmness during the ripening and thereby affecting postharvest quality of fruits. These degrading enzymes include polygalacturonase (PG), pectin methyl esterase (PME), pectin esterase (PE) and cellulase. They play an important role in softening the fruit. PG is the principal enzyme involved in the hydrolytic cleavage in pear, avocado and apples [37]. Activities of cell wall degrading enzymes such as PE, PME and PG are suppressed by PAs leading to maintenance of firmness in fruits along with enhancement of shelf life. Therefore, role of PA in reducing the softening of fruit was reported due to the inhibitory effects of PAs on the enzymes (PG, PE and PME) involved in degradation of cell wall [9]. There are numerous reports referring to improvement of firmness in fruits by application of Pas (Table 1). Fruit commodities includes apricot [18], citrus [38], peaches [39], plums [28], pomegranates [30], and strawberries [33]. PUT (1 mM) treatment in “Hayward” kiwifruit showed an increase in firmness of flesh [22]. Mango cv. Kensington Pride showed enhanced fruit firmness after being exogenously treated with PUT (2 mM) [24]. The combination of PUT and the ultrasound treatment also resulted in reduction in softening of peach fruits under storage and highest firmness values were recorded for PUT and ultrasound treated fruits [40]. Cationic nature of PAs and their interaction with negatively charged carboxylic group led to induction of firmness in fruits thereby improving the quality of fruits at postharvest stage [40]. Effect of exogenous application of PUT (0.5, 1.0, 1.5 mM) and SPD (0.5 mM) was studied under storage conditions in “Flame Seedless” grapes. The PME activity in untreated stored berries increased till 45 days after storage and reached peak at 60 days whereas in berries treated with PAs there was a significant decrease in PME activity. PUT was found less effective than SPD in suppressing activity of PME. The SPD when applied at lower concentrations of 0.5 and 1.0 mM suppressed PME activity till 60 d after storage whereas higher concentration of SPD (1.5 mM) led to increased activity of PME as similar to the control till 45 days of storage. PUT 0.5 mM was found effective in suppressing the PME activity in comparison to higher concentrations of 1.0 mM and 1.5 mM [21]. SPE 1.5 mM and SPD 2.0 mM showed reduced activity of PG and then followed by lipoxygenase (LOX) activity in kiwifruit [23]. Pre- and post-harvest application of PUT (0.1, 1.0 or 2.0 mM) to “Angelino” plums resulted in delayed fruit ripening under storage (0 ± 1 °C, 90 ± 5% RH). Lower activity of exo-PG enzyme was reported from treated fruit pulp and skin tissues under storage. Endo-PG activity was also found to be reduced in fruits in which PUT was sprayed at preharvest stage, whereas postharvest applications failed to affect the endo-PG activity. PE and EGase activity in fruit skin and pulp tissues was affected by both pre- and post-harvest PUT treatments [28].

#### 3.2.3. Effect on Mitigation of Mechanical Damage

Mechanical damage is one important aspect which affects the physico-chemical quality attributes of fruits in postharvest handling and makes them prone to the attack of microbes, insects and pests and therefore ultimately spoils the produce under storage. Apricots when treated with PUT (1 mM) after 24 h by pressure infiltration led to retention in firmness and when subjected to a force of 25 N equatorially on fruit zones and were stored (90% RH, 10 °C) for six days enhanced the resistance towards the mechanical damage and were evaluated in terms of low deformation percentage, followed by lowered bruising volume [18]. It was due to the enhanced firmness of fruit because of electrostatic interaction of PUT with the fruit tissue which ultimately provided firmness to fruit [18]. Low mechanical damage and reduced flesh deformation was observed in PUT (1 mM) treated “Blackstar” plum fruits. The accumulation of cell wall conjugated PUT and SPD was observed in plums for two weeks under storage. Enhanced levels of bounded PAs contributed to the high level of firmness. Besides this, during initial seven days of storage, damaged control plums showed higher levels of free SPD and were considered a physiological marker for stress induced by mechanical damage [27]. PA application was found to be effective against mechanical damage in climacteric and non-climacteric fruits and therefore enhanced the shelf life and other quality attributes [9]. PA treatments could prove to be a solution to reduce the mechanical stress and their after effects in fruits during storage at postharvest stage (Table 1).

### 3.3. Effect on Ripening Inducing Factors

#### 3.3.1. Effect on Ethylene Biosynthesis

Ethylene evolution is one of the important event during postharvest phase. Ethylene evolution is inversely proportional to shelf life of fruits. The metabolic processes are driven by enzymes and most of the biochemical processes involves the active participation of enzymes. The arginine decarboxylase (ADC) activity is influenced by the exogenous application of SPD and also affects the enzymes involved in the biosynthesis of ethylene, lead to delay in senescence of fruit [9]. PAs are reported to be anti-ethylene in nature though both having the same precursor, i.e., SAM [41], and is supported by others [9,21,27,33,41,42] (Table 1). Application of PUT (1 mM) on mango (cv. Kensington Pride) under ambient conditions at pre-harvest stage resulted in lowered synthesis of ethylene and prolongation in ripening of fruit [36]. Increase in the concentration of free PAs in pulp and skin was studied and the skin possessed around 55.5% high levels of PAs in comparison to pulp [24], whereas 1 mM SPM treatment in mango (cv. Kensington Pride) fruit was reported to be most effective in reducing the production of ethylene (1.18 nM/kg/h) [24]. This efficacy of SPM emphasizes the application of SPM in prolonging shelf life of mango. Treatment of “Angelino” plums with PUT (1 or 2 mM) led to a significant reduction in ethylene synthesis [29]. Low pressure infiltration of PUT (1 mM) application reduced ethylene biosynthesis in apricots [18]. Similar results were obtained in strawberry cv. Selva where PUT (2 mM) application led to a significant reduction in production of ethylene [33]. In Kiwifruit cv. Hayward, exogenous treatment with PUT (1 mM) inhibited the production of ethylene [22]. Combination of PUT along with carnauba wax acted as a physical barrier and resulted in reduced ethylene production in pomegranates under storage for around 30 days [32]. The rationale behind the reduced reduction of ethylene production after the application of carnauba wax was the reduction in the interchange of gases due to the reduced respiration [43] and therefore when applied in combination with PAs synergistically reduced the production of ethylene gas. The above studies led to the conclusion that the PAs are effective in inhibition of ethylene release from the fruits.

#### 3.3.2. Effect on Respiration

Respiration plays an important role in diverse physiological processes till the senescence phase and is vitally involved in diverse postharvest losses. Postharvest application of PAs has led to the reduction in respiration rate in different kind of fruits (Table 1). Application of PUT (1 mM) was reported to significantly reduce the respiration rate under storage conditions when applied on apricots [18] and kiwifruit [22]. The reduction in respiration rate and fruit softening in mango was reported with PUT and SPD treatments (0.01, 0.5 or 1 mM) [24]. Reduced respiration rate of pomegranates were observed when treated with PUT and SPD (1 mM) by different application methods (pressure infiltration or immersion) [30]. Plums (cv. Angelino) when stored under low temperatures (0 ± 1 °C) and followed by the PUT application resulted in delaying respiration followed by an improvement in fruit quality [29]. PAs were also applied in combination with other treatments. The synergistic impact of the combination of PUT and carnauba wax pre-treatment in pomegranates reduced the respiration rate when pomegranates were stored under cold storage conditions for a period of 60 days [32]. Similar, results were noticed when PUT treatment along with ultrasound led to the inhibition of respiration rate and also delayed the process of ripening in peaches [40].

### 3.4. Effect on Biochemical Attributes, Antioxidant Compounds and Antioxidant Enzymes

#### 3.4.1. Effect on Total Soluble Solids

Iranian apricots namely “Lasgerdi” and “Shahrodi” were treated with PUT 1.0, 2.0, 3.0 or 4.0 mM followed by packaging in containers with covers made of polyethylene and stored at 4°C, 95% RH for 20 days. A decrease in TSS was reported in fruits treated with PUT in comparison to control [20] and was supported with the results reported in apricots cv. Tokhm Sephid and in peaches cv. Zapherani [44]. When berries of “Flame Seedless” grapes were treated with PAs, TSS showed an increasing trend in control berries under storage up to 45 days in comparison to berries treated with PAs. During 45–60 days, declination in TSS was found in both treated and control berries. In control, the TSS was reported to be decreased by 1.4% whereas in the berries treated with PUT 0.5, 1.0 or 1.5 mM, reduced by 1.1, 0.3 and 0.4%, respectively [21]. “Flame Seedless” berries exposed to the exogenous treatment of SPE 1.0 or 1.5 mM and stored under cold store conditions showed an increasing trend in TSS up to 45 days under storage and found to decrease during the rest of period under storage. Whereas, SPE 1.0 mM treated berries showed highest content of TSS in contrast to the berries treated with the lowest (0.5 mM) SPE concentration [35]. The exogenous application of PUT 1.0 mM, SPE 0.01 mM, SPD 0.5 mM on mango cv. Kensington Pride resulted in significant increase in mean TSS with PUT treatment, whereas in case of SPE the mean TSS decreased as compare to control [36]. In another study, exogenous application of 2.0 mM PUT in “Langra” mango led to the highest acidity and provided the good blend of TSS and acidity under storage (13 °C, RH 90–95%) [25]. Similarly, in various other fruits PUT treatment resulted in decreased TSS content in comparison to the fruits without treatment [27,28,29,40,44] (Table 1).

#### 3.4.2. Effect on Browning

Fruit pericarp browning in postharvest is one of the critical problems affecting the marketability of fruits. Mostly the mechanically damaged fruits are highly prone towards browning due to the intervention of diverse enzymatic reactions and due to enhanced metabolism in fruit. The physiological effects that take place in the damaged fruits are juice leakage, browning of flesh and loss in weight [45]. Anti-browning effect of exogenously applied PUT (1 mM) was shown by mechanically damaged plums during their packaging and handling and therefore, browning was reduced [27]. Study was supported by anti-browning effect of exogenously applied PAs in pomegranates [30]. Peach fruits treated with PUT (1 mM) alone or in combination with ultrasound (32 kHz at 60 W/L, 10 m) showed reduced symptoms of browning and leatheriness as compare to control fruits after three weeks under storage [40]. Oranges when treated with PUT (5 mM) and methyl jasmonate (10 µM) upto four months under storage showed a significant reduction in browning of fruits in comparison to controls [46]. When PA is applied on the apricot fruits kept under the cold storage, it strengthens the cell walls and kept PPO and TP in separate compartments resulting in the mitigation of browning of fruits under the cold storage conditions [19]. Therefore, the application of PAs could be commercially used to mitigate the postharvest browning of fruits to improve the marketability and maintain their quality attributes (Table 1).

#### 3.4.3. Effect on Antioxidant Compounds and Antioxidant Activity

AA oxidation takes place under the storage conditions of fruits and therefore the levels of AA decreases under storage conditions. This reduction could be due to the oxidative conversion of dehydroascorbic acid to diketogluconic acid [47]. PAs plays an important role in mitigating the activity of ascorbate oxidase. The application of PUT (1, 2, 3 or 4 mM) on apricot cv. Lasgerdi and Shahrodi when stored under a temperature of 4 °C and RH 95% for 20 days showed a significant difference in the levels of AA in PA treated fruits and the untreated fruits. The minimum loss of AA was observed with 4 mM PUT treatment [20]. 

PAs play an important role in maintaining the total phenolic content (TPC) in fruits under storage conditions (Table 1). Breakdown of cell structure during storage leads to a fast decrease in TPC [48]. The effect of postharvest application of PUT on maintaining the levels of TPC was studied on apricot cv. “Lasgerdi” and “Shahrodi” at 4 °C storage. TPC were reported to be maintained at maximum levels when 4 mM PUT was applied in comparison to other concentrations (3 mM or 2 mM) [20]. Significant increase in TPC after PUT treatments (1.0 or 2.0 mM) was also observed in “Samar Bahisht Chausa” mangos under 28 days storage at 11 ± 1 °C [26]. Similar results were reported when other apricot cultivars “Lasgerdi” and “Shahrodi” were treated with PUT (4 mM) and were stored at 4 °C. Highest antioxidant activity was reported in the treated fruits whereas control possessed lowest antioxidant activity [20]. In kiwifruit cv. Allison, the exogenous application of PAs viz. SPE (0.5, 1.0, 1.5 mM) and SPD (1.0, 1.5, 2.0 mM) was done using immersion method. TPC followed a decreasing trend during storage due to the suppressed activity of PPO. Decrease in activity was due to reduced respiration rate due to PAs treatment. The results showed the action of PAs as perseverant of precursors responsible for antioxidant activity i.e., diverse pigments of plant origin, phenols and diverse vitamins. The application of 1.5 mM SPE proved to be best in infusing and higher antioxidant capacity (31.0 µM Trolox/g) whereas SPD 2 mM treated fruits showed (30.0 µM Trolox/g) [23]. “Samar Bahisht Chausa” mangos were treated with different concentrations (0.0, 0.1, 1.0 or 2.0 mM) of PUT and were made to ripen for seven days at the temperature range of 32 ± 2 °C followed by their storage at 11 ± 1 °C up to 28 days. Result showed a significant increase in antioxidant contents after treatment with PUT [26]. Results of all the studies in different fruits corroborate each other and dominantly emphasises the enhanced activation of the antioxidant levels in the fruits after their exogenous treatment before storage.

Antioxidant activity in fruits is the expression of diverse class of secondary metabolites. Antioxidant property of fruits is biochemically facilitated by the presence of compounds such as flavanoids, TPC, and AA. These antioxidants are nutritional from the health point of view and ensuring their bioavailability under storage conditions is a challenge. It is sine qua non to maintain the levels of antioxidants in fruits under storage by up keeping the overall attributes required for fruit quality. The decline in the contents of antioxidants under colder temperatures leads to the suppression of active oxygen species (AOS) content [49]. Under chilling temperature the lipid peroxidation of the susceptible membranes occurs followed by their degradation and senescence is universally acceptable phenomenon [50]. Antioxidant system consisting superoxide dismutase (SOD), catalase (CAT) and peroxidase (POX) are involved in scavenging of free radicals [51].

Fruit ripening is considered as an oxidative phenomenon and follows an increase in the levels of AOS, hydrogen peroxide and superoxide anions [52]. The natural balancing mechanism of elimination of AOS by an active antioxidant system is one of the most powerful system [53]. By the action of regenerated lipid phase antioxidant viz. α-tocopherol [54] controls the prominent metabolic bioconversions during ripening viz. cell division [55,56,57,58], cell wall expansion [59] and organogenesis [60]. The fundamental role of antioxidative system consisting CAT, SOD and ascorbate glutathione during the ripening of tomato reduced the oxidative damage at breaker stage. Rationale being an increase in the levels of antioxidants in the aqueous phase which in turn affect the redox state of metabolites during ripening. At molecular level the mRNA transcripts encoding these oxidative enzymes increased their levels at breaker stage, highest level at pink stage followed by their reduction during ripening [61].

#### 3.4.4. Effect on Antioxidant Enzymes

Activity of enzymes involved in fruit ripening was greatly suppressed by exogenous application of PAs in “Kensington Pride” mangoes [36]. Treatment of Valencia oranges with PUT (5 mM) significantly lowered the lipid peroxidation [46]. Antioxidant system of treated apricot fruits modulated simultaneous increasing levels of antioxidative enzymes SOD, CAT and POX which scavenges the ROS formed under the chilling stress. The SOD activity in apricot cv. Bagheri first increased and after some time it became stable, whereas in SPD treated fruits, activity of SOD enhanced. In contrast, in case of cv. Asgharabadi, the enzyme activity increased for 7 days continuously and then decreased. Besides this, PA treated fruits especially SPD showed increased SOD activity over the control. CAT activity also decreased during storage, whereas PUT treated cv. Bagheri showed enhanced activity of CAT. Enhanced POX activity was reported in both cultivars when fruits were treated with SPD. PPO activity found in control fruits of cv. Bagheri showed an increase during storage whereas there was decrease in activity of PPO in cv. Asgharabadi. The PPO activity was also reported to be slowed down in PA treated fruits [19]. In zucchini, PUT (1 mM) applied exogenously by immersion to fruits for 20 min at 20 °C and stored at low temperature led to an increased activity of LOX in fruits under chilled conditions deters the lipid peroxidation and thereby resists the impact of CI. Besides this the increment in the activities of ascorbate peroxidase (APX), CAT and glutathione reductase (GR) led to scavenging of ROS and therefore resulted in enhanced shelf life under low temperature storage [34]. All of the above studies on exogenous application of PAs have modulated the diverse range of enzymes that contributed to enhancement of shelf life and improved other quality attributes of fruits (Table 1).

### 3.5. Effect on Chilling Injury

**Figure 2:**
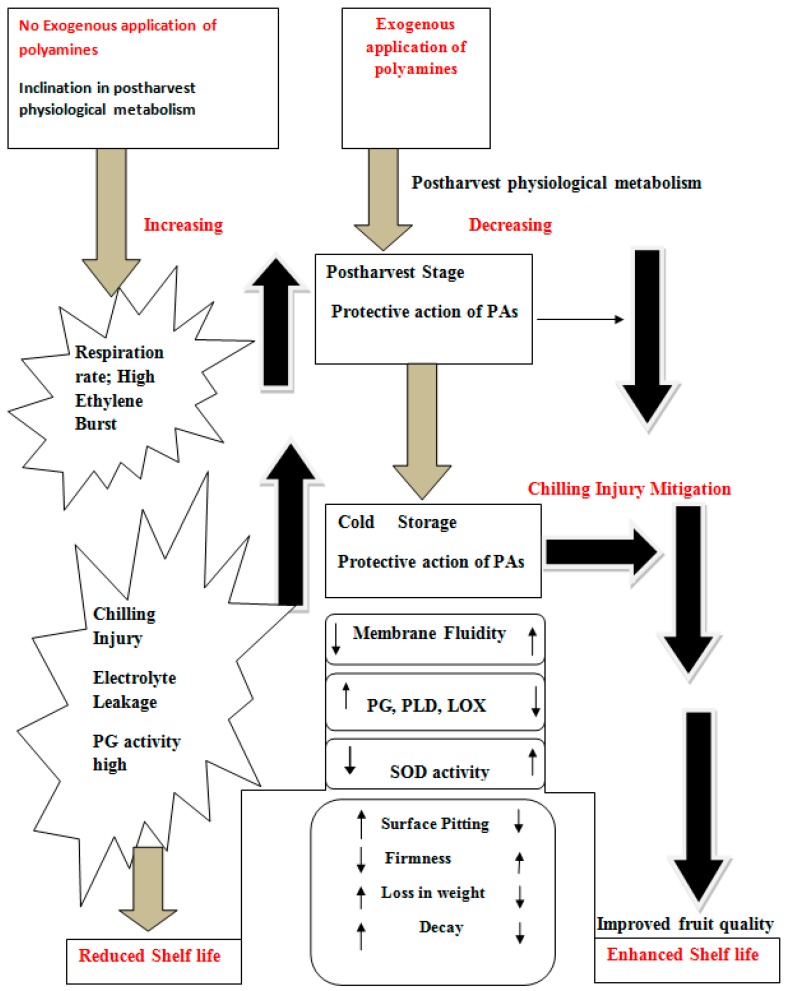
Possible actions of exogenously applied polyamines on fruits and mitigating CI and improving shelf life; PG, Polygalacturanase; SOD, Superoxide dismutase; LOX, Lipoxygenase; PLD, Phospholipase D; Decreasing trend (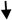); Increasing trend (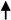).

**Figure 3:**
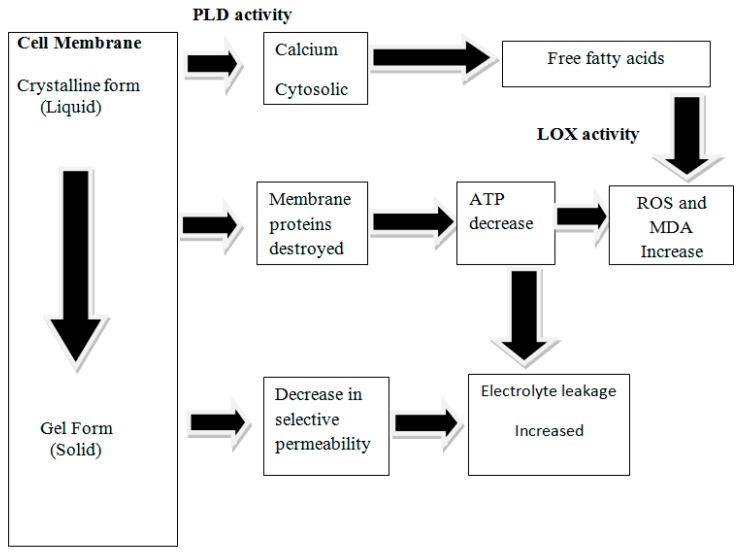
Mechanism of Chilling stress causing chilling injury: PLD, Phospholipase D; LOX, Lipoxygenase; ATP, Adenosine triphosphate; ROS, Reactive oxygen species; MDA, Malondialdehyde.

CI is the most common physiological disorder occurred in tropical and subtropical fruits postharvest storage. Postharvest CI in tropical and subtropical fruits could be mitigated by the exogenous application of PAs. Figure 2 shows the impact of chilling stress on physiology of fruits. Their important role in modulating gene expression and encountering the diverse stresses prove their importance in plant physiology and other growth processes. Mechanism of PAs to mitigate CI is explained in Figure 3. PA-led positive fluctuation in postharvest physiological metabolism of highly perishable fruits is a future key tool to prolong the shelf life.

Subtropical and tropical fruits are highly prone to CI when exposed to temperature range of less than 10 °C [62]. As a result of CI, cell organelles and membrane damages leading to the leakage of solutes followed by an increase in the content of hydrogen peroxide and malondialdehyde (MDA). PAs play an important role in providing firmness of cell walls, maintaining structure of cell membranes, providing resistance to leakage, and inhibiting the development of ROS [63]. Application of SPE, SPD and PUT (0.5, 1.0 or 1.5 mM) on sweet lemons at 3 and 10 °C reduced CI significantly [64]. In Valencia oranges, exogenous application of PUT (5 mM) led to the reduction in CI and increased tolerance towards low temperature injury [46]. Similarly, in Valencia orange var. Olinda, treatment with SPD (1.5 mM) led to reduction in CI when stored under low temperature [65]. Pre-storage pressured infiltrated application to pomegranates with SPD and PUT (1 mM) significantly reduced skin browning by ~30% as compare to ~55% in control fruits [30]. PUT, SPE and SPD (1 mM) when applied exogenously on “Sinatra” zucchini by way of immersion for 20 min at 20 °C were also effective on reducing CI during storage at (4 °C, 85–90% RH) for 3, 5, 10 or 14 days. In addition, treated fruits showed decreased CI and hydrogen peroxide contents. PUT was found best in mitigating thio-barbituric acid-reactive substances (TBARS) and hydrogen peroxide generated under the cold stress [34] (Table 1).

## 4. Tomato Transgenics Showing Over-expression of Genes Encoding PAs for Maintaining Quality and Shelf Life

The expression of *S*-adenosylmethionine decarboxylase (ySAMdc) gene from yeast fused with E8 promoter, which is ripening inducible, led to rise in PA levels particularly SPE and SPD in tomato fruit [66]. Conversion of PUT to higher PAs is reported due to enhanced expression of ySAMdc. Results showed a remarkable improvement in juice quality, phytonutrient contents and enhanced the life of vine [10]. Development of transgenic tomatoes with integrated E8-ySAMdc chimeric gene into the genome of tomatoes facilitated the accumulation of PAs viz., SPD and SPE and also led to the rise in accumulation of organic acids, glutamine and asparagines in red tomato fruit. Study reported an improvement in flavour, increased acid to sugar ratio, followed by accumulation of choline, which is one of the vital amine playing a significant biological role [11].

Transcriptomic analysis followed by metabolite profile study in genetically engineered tomato with ySAMdc to produce SPE and SPD showed that PAs act as anabolic growth regulators [67]. Transgenic plants of tomato with ySAMdc gene from Sacchromyces cerevisiae led to 1.7–2.4-fold higher endogenous production of SPE and SPD leading to inherent tolerance against high temperature stress [68]. Expression of spermidine synthase (ySpdSyn) gene from yeast with constitutive promoter as CaMV35S along with E8, a fruit ripening promoter in tomato, was evaluated for their impacts on fruit physiology. It was reported that the constitutive expression of ySpdSyn gene led to an increase in levels of SPD both in leaves and in developing fruit, whereas expression of E8-ySpdSyn facilitated the accumulation of SPD during early and fruit ripening stages. In transgenic fruits, developed in transgenic lines of tomato possessing,ySpdSyn gene was reported to induce long life, and decrease shrivelling and decay in comparison to the wild type tomato fruits [69]. Another study reported that tomato fruit engineered with ySAMdc gene in combination with fruit ripening E8 promoter showed an inverse relationship between the synthesis of higher PAs and ethylene evolution while ripening, which led to increased shelf life of tomato fruit [66]. Table 2 is representing the transgene constructs encoding PA synthesis with suitable promoters to enhance endogenous polyamine levels in tomato.

**Figure 4:**
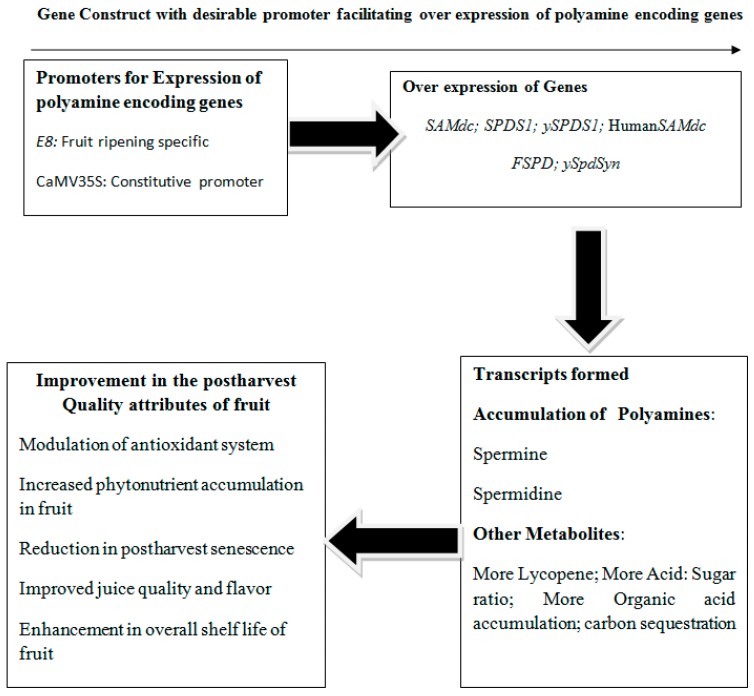
Scheme showing the over expression of transgenes encoding polyamines with suitable promoters for improving quality attributes and shelf life of horticulture crops.

Introgression of genes encoding higher level PAs traits when incorporated in tomato using the transgenic technology led to suppression of ethylene release ultimately reported to combat the negative impacts of ethylene. The investigation of the effect of transgenes encoding PA production metabolic profiling which proved that changes related to ripening in fruit were both ethylene dependent and independent. The metabolome of fruit was found to be controlled by regulators such as ethylene and PAs [70]. ySpdSyn gene which encodes biosynthesis of PAs (SPDS1) when overexpressed led to 1.5 to 2.0 fold increase in the content of PAs. Genes encoding lycopene biosynthesis i.e., phytoene desaturase, phytoene synthase and deoxy-D-xylulose 5-phosphate synthase showed upregulation in fruits which were ripened. Besides this the genes responsible for lycopene degradation viz. lycopene beta cyclase and lycopene-epsilon cyclase got down regulated and as a result fruit possessed higher amount of accumulated lycopene in tomato fruit [71]. The genetic modification of tomato fruit with h-SAMDC (Human-SAMDC) gene driven by a fruit specific promoter (2A11) led to an overexpression of this gene followed by reduction in ethylene production by 50%, and the fruit ripening on-vine was delayed by 11 days as compared to wild type fruits. Overall, result showed an increased levels of PAs such as PUT due to an inter conversion of SPD/SPM to PUT in the transgenic lines by the mechanism of acetylation followed by enhanced levels of vitamin C, TSS and lycopene in transgenic tomato fruits as compare to wild type fruits [72]. Figure 4 highlights the genes encoding PA biosynthesis with suitable promoters representing the accumulation of PAs at large levels. Therefore, development of transgenic fruits and vegetables over expressing the genes encoding PAs is a novel mechanism which can act as a potent tool to enhance shelf life. Synergistic association of fruit breeding techniques and biotechnology for development of transgenic fruits and vegetables enables the inherent mechanism of biosynthesis of PAs to modulate anti-oxidative mechanism which will pave the way to acquire tolerance against diverse kinds of stresses destroying quality attributes and shelf life of fruits.

## 5. Conclusions

PAs, being biodegradable and environment friendly organic compounds, will promote sustainability by mitigating the postharvest losses of fruits. Stage of application and optimisation of concentrations of these compounds are of utmost importance and of great consideration when exogenously applied. Their application at pre- and post-harvest phases will make a difference by mitigating the biotic and abiotic stress in horticultural crops as well. However, during the beginning of postharvest phase, sudden decline in PAs shortens shelf life of fruit due to diverse biotic and abiotic stress under storage environment. Besides this, PAs could be produced in situ by the application of emerging technologies such as genetic engineering by incorporating the genes encoding PAs. These genes must be incorporated with suitable promoters to facilitate in situ biosynthesis of PAs at higher levels. Such a biotechnological interventions will pave the path for developing an inherent mechanism for in situ endogenous production of PAs in fruits and vegetables and will improve the fruit quality and shelf life under storage. Besides this, for exogenous applications, nanotechnology based carriers could be developed for the targeted assimilation of the PAs when used for the shelf life enhancement in perishable horticultural crops.

## Figures and Tables

**Table 1:** Summary of effect of PAs on postharvest physiology and quality of fruits.

Fruit	Polyamine	Doses	Effects	Reference
Apricot	PUT	1 mM	Increased shelf life, reduced respiration rate, weight loss, ethylene biosynthesis, lowered cellular juice leakage and browning	[18]
	PUT	4 mM	Reduced weight loss	[48]
	PUT or SPD	1 mM	Tolerance to CI at 1°C for 21 days, reduction in ethylene release, stabilisation in colour and firmness of fruits	[19]
	PUT	4 mM	Reduction in weight loss, maintain firmness, delay ripening	[20]
Grapes cv.Flame Seedless	PUT or SPD	0.5 mM	Maintained berry firmness, peel colour and anthocyanins, reduced electrolyte leakage, PME, decay incidence, degradation of TSS and TA and enhanced shelf life and low temperature storability	[21]
Kiwifruit	PUT	1 mM	Reduction in ethylene evolution	[22]
	SPM&SPD	1.5, 2.0 mM	Low rate of respiration, diminished PGand LPX activities, enhanced shelf life	[23]
Mango	SPM	0.5 mM	Reduction in weight loss, decrease in softness, decrease in respiration rate	[36]
	SPM and PUT	0.5, 1 mM	Reduced weight loss, reduced fruit colour	[24]
	PUT	2.0 mM	Reduced physiological weight loss, improved blend of TSS, acidity and palatability rate	[25]
	PUT	2.0 mM	Inhibition of ethylene release, respiration rate, fruit softening, suppression of cell wall enzymes *endo*, *exo*-PG and *EGase*, modulation of antioxidant enzymes: SOD CAT, POX, overall fruit quality maintained	[26]
Plum	PUT	1 mM	Increased firmness peel and flesh firmness, decreased ethylene and CO_2_ evolution. SPD acted as physiological marker against mechanical damage	[27]
	PUT	1 mM	Reduction in ethylene release and soluble solids, increased fruit and flesh firmness, and extended storability	[28]
Pomegranate	PUT or SPD	1 mM	Maintained fruit firmness, enhanced shelf life, reduced husk scald, prevented skin browning	[31]
	PUT or SPD	1 mM	Heat treatment induced PA induction, reduction in fruit softening, CI mitigation	[30] *
	PUT + Carnauba wax	2 mM Ratio (1:10)	Low respiration rate, ethylene release and CI, no discoloration of fruit peel, fruit firmness maintained, mitigation in pitting surface	[32]
Strawberry cv. Selva	PUT	1 mM 2 mM	Reduced weight loss and ethylene release and maintained firmness	[33]
“Valencia” oranges	PUT + Methyl jasmonate	5 mM + 10 µmol	Lowered enzymatic activity and chilling injury	[46]
	SPD	1, 1.5 mM	Maintained TSS, tritatable acidity, flavour index	[65]

Note: * PAs induced by heat treatment.

**Table 2:** Transgenic constructs encoding polyamine synthesis with suitable promoters to enhance endogenous polyamine levels in tomato.

Tomato Gene Constructs Encoding Polyamines with Promoters	Observations	Reference
*ySAMdc-E8*	Levels of SPE and SPD increased and accumulated; *ySAMdc* resulted in expedited conversion of PUT to the higher PAs; lycopene increased; vine life prolonged; improved juice quality from fruits.	[10]
*ySAMdc*(Ripening targeted)	Increased levels of SPE and SPD; differential gene expression showed that majority of genes were up-regulated in the fruits and possessed higher levels of PAs; endogenously produced PAs acted as anabolic growth regulators.	[67]
*ySAMdc-E8*	Inverse relationship between PAs and ethylene during ripening of fruit; expression of *ySAMdc* modulated the inverse relationship between higher PAs and ethylene; decline in SPE and SPD could be mitigated without any alteration in ethylene biosynthetic pathway.	[66]
*E8-ySAMdc*	Accumulation of PAs specifically SPE and SPD; fruit cells perceived SPE and SPD as organic nitrogenous signalling metabolites which facilitated synthesis of organic acids, asparagine and glutamine in red coloured fruits; led to sequestration of carbon, enhanced ratio of acid to sugar, fruit flavour; synthesis of essential amine chlorine important for biological functioning of brain; inferred PAs as anti-apoptotic in nature.	[11]
*ySpdSyn* expressed with constitutive promoter *CaMV35S* and ripening specific promoter *E8*	Higher lycopene accumulation; reduction in postharvest senescence; reduction in spoilage	[69]
*E8-ySpdSyn*	Accumulation of SPD during ripening of fruit	[69]
*SPDS1*	The over expression of spermidine synthase a gene encoding SPE in the transgenic tomato led to the synthesis of lycopene followed by its accumulation in fruit.	[71]
*Human SAMdc* and promoter specific to fruits (*2A11*)	Accumulation of higher PAs such as SPE and SPD to increased levels; reduction in the evolution of ethylene gas by 50%; increase in the amount of vitamin C, TSS and lycopene.	[72]

## Abbreviations

AA	Ascorbic acid
ACC	1-aminocyclopropane-1-carboxylic acid
ADC	Arginine decarboxylase
AIH	Agmatimeimino hydrolase
AOS	Active oxygen species
APX	Ascorbate peroxidise
ATP	Adenosine triphosphate
CAT	Catalase
CI	Chilling injury
GR	Glutathione reductase
LOX	Lipoxygenase
MDA	Malondialdehyde
ODC	Ornithine decarboxylase
PAs	Polyamines
PE	Pectin esterase
PG	Polygalacturonase
PLD	Phospholipase D
PME	Pectin methyl esterase
POX	Peroxidise
PPO	Polyphenol oxidase
PUT	Putrescine
ROS	Reactive oxygen species
SAM	*S*-adenosyl methionine
SOD	Superoxide dismutase
SPD	Spermidine
SpdS	Spermidine synthase
SPE	Spermine
SpeS	Spermine synthase
TA	Titrable acidity
TBARS	Thio-barbituric acid-reactive substances
TPC	Total phenol content
TSS	Total soluble solids
ySAMdc	*S*-adenosylmethionine decarboxylase
